# Cancer-Related Causes of Death among HIV-Infected Patients in France in 2010: Evolution since 2000

**DOI:** 10.1371/journal.pone.0129550

**Published:** 2015-06-17

**Authors:** Marie-Anne Vandenhende, Caroline Roussillon, Sandrine Henard, Philippe Morlat, Eric Oksenhendler, Hugues Aumaitre, Aurore Georget, Thierry May, Eric Rosenthal, Dominique Salmon, Patrice Cacoub, Dominique Costagliola, Geneviève Chêne, Fabrice Bonnet, the ANRS EN20 Mortalité 2010 study group

**Affiliations:** 1 INSERM, ISPED, Centre INSERM U897-Epidémiologie-Biostatistiques, Bordeaux, France; 2 Université Bordeaux, ISPED, Centre INSERM U897-Epidemiologie-Biostatistiques, Bordeaux, France; 3 Service de médecine interne et maladies infectieuses, CHU de Bordeaux, Bordeaux, France; 4 Service de Maladies Infectieuses, Hôpital Brabois, Vandoeuvre-Les-Nancy, France; 5 Service d’Immunopathologie Clinique, Hôpital Saint Louis, AP-HP, Paris, France; 6 Service des Maladies Infectieuses, Centre Hospitalier, Perpignan, France; 7 Service de Médecine Interne, Hôpital de l’Archet, Nice, France; 8 Université de Nice-Sophia Antipolis, Nice, France; 9 Unité de Pathologie Infectieuse, Pôle Médecine, Hôpital Cochin, AP-HP, Paris, France; 10 Université Paris Descartes, Paris, France; 11 Service de Médecine Interne, Hôpital La Pitié-Salpêtrière, AP-HP, Paris, France; 12 Université Pierre et Marie Curie, Paris, France; 13 INSERM, UMR_S 1136, Institut Pierre Louis d’Epidémiologie et de Santé Publique, Paris, France; 14 Sorbonne Universités, UPMC Université Paris 06, UMR_S 1136, Institut Pierre Louis d’Epidémiologie et de Santé Publique, Paris, France; Rollins School of Public Health, Emory University, UNITED STATES

## Abstract

**Objectives:**

The current study aimed at describing the distribution and characteristics of malignancy related deaths in human immunodeficiency virus (HIV) infected patients in 2010 and at comparing them to those obtained in 2000 and 2005.

**Methods:**

Data were obtained from three national surveys conducted in France in 2010, 2005 and 2000. The underlying cause of death was documented using a standardized questionnaire fulfilled in French hospital wards involved in the management of HIV infection.

**Results:**

Among the 728 deaths reported in 2010, 262 were cancer-related (36%). After a significant increase from 28% in 2000 to 33% in 2005 and 36% in 2010, cancers represent the leading cause of mortality in HIV infected patients. The proportion of deaths attributed to non-AIDS/non-hepatitis-related cancers significantly increased from 2000 to 2010 (11% of the deaths in 2000, 17% in 2005 and 22% in 2010, p<0.001), while those attributed to AIDS-defining cancers decreased during the same period (16% in 2000, 13% in 2005 and 9% in 2010, p = 0.024). Particularly, the proportion of respiratory cancers significantly increased from 5% in 2000 to 6% in 2005 and 11% in 2010 (p = 0.004). Lung cancer was the most common cancer-related cause of death in 2010 (instead of non-Hodgkin lymphoma so far) and represented the leading cause of death in people living with HIV overall.

**Conclusions:**

Cancer prevention (especially smoking cessation), screening strategies and therapeutic management need to be optimized in HIV-infected patients in order to reduce mortality, particularly in the field of respiratory cancers.

## Introduction

The advent of combination antiretroviral therapies (cART) has dramatically reduced mortality and incidence of AIDS-defining events in people living with HIV [1]. However, the risk of malignancies, including non-AIDS-related cancers, remains higher in patients living with HIV than in the general population, with a poorer prognosis reported [2–4]. Among them, the risk of lung cancer is two to three time higher in HIV-infected patients than in the general population [3]. Lung cancer is a leading cause of mortality in HIV-infected patients [5] with a worse prognosis than in the general population [6–8]. Smoking represents an important risk factor in the development of lung cancer in HIV-infected patients [9] with higher smoking rates in this population than in the general population.

In two previous national prospective surveys specifically designed to assess the underlying cause of death in HIV-infected patients conducted in 2000 and 2005 in France [10,11], we showed that in these two periods of time, malignancies accounted for 28% and 33% of the causes of death in this population, respectively, and the proportion of non-AIDS-related cancers significantly increased from 11% in 2000 to 17% in 2005 [5].

The objectives of the present study were to describe the distribution and characteristics of malignancy related deaths in patients living with HIV in 2010 as well as assessing changes from 2000 and 2005 in a country where care and treatment are available and free for all persons living with HIV.

## Materials and Methods

The Mortalité 2010 survey succeeds to two similar surveys (Mortalité 2000 and Mortalité 2005), which respectively included 185 and 341 hospital wards providing care and treatment to HIV-infected patients in France [10,11]. In order to simplify logistics, the 2010 survey was conducted within centres reporting at least five deaths (regardless the cause of death) during one of the previous surveys. Thus, 90 centres participated in the survey [12]. The distribution of the deaths observed in 2005 in these 90 centres was not statistically different from the distribution observed in the 341 participating centres. Each participating centre had to report all deaths among HIV-infected patients that occurred in 2010 in their hospital, or among patients followed-up in their department but who died in another institution or at home. Physicians in charge of the patients completed an online standardized questionnaire (dedicated website). Coherence of data collected was secondarily validated by two physicians specialized in HIV clinical research. The Epidemiology Centre on Medical Causes of Death (INSERM, CépiDc) determined the underlying cause of each death according to the rules of the International Classification of Diseases- 10th revision, based on the questionnaire data on causes of death and morbidity. The underlying cause of death was defined as the disease or injury, which initiated the train of morbid events leading to death and was validated according to patient’s file by a medical committee. In order to allow comparisons between the 3 Mortalité surveys, we used the same algorithm for determining the underlying cause of death in 2010 as in 2000 and 2005 [11,12], rather than the more recent Coding Causes of Death in HIV (a standardized method for coding the underlying cause of death in HIV-infected persons which has been recently implemented in international HIV cohort mortality studies [13]).

Cancers were classified as AIDS-related when active pathology at time of death included one AIDS defining cancer according to the classification of the Centers for Disease Control and Prevention for HIV infection as revised in 1993 [14]: high grade non-Hodgkin lymphoma (NHL), Kaposi sarcoma and cervical cancer. Other cancers were classified as either hepatitis-related cancer (defined as the occurrence of a liver cancer in HIV-infected patients co-infected with hepatitis C virus [HCV] and /or hepatitis B virus [HBV]) or non-AIDS/non-hepatitis (NANH)-related cancer. Hepatitis status was determined according to the test results for HCV (positivity of anti-HCV antibodies or HCV RNA) or HBV (positivity of HBV surface antigen or HBV DNA).

Comparisons of categorical variables between groups were performed using the χ2 test or χ2 corrected, or Fisher's exact test, depending on the actual expected values under the hypothesis of independence. Comparisons of continuous variables were performed with Student's t-tests (comparison of means) or the Wilcoxon test (comparison of distributions). The distribution of cancer-related causes of death was compared between 2000, 2005 and 2010, and between 2005 and 2010 using a multinomial logistic model adjusting for gender and age. All analyses were performed using the 9.1.3 service pack 2 version of SAS software (SAS Institute Inc., Cary, NC, USA).

## Results

A total of 728 deaths were reported by the 90 centres in 2010 (836 for these same centres in 2005 and 783 in 2000) among approximately 82,000 HIV-infected patients with follow-up in 2010. The median age at death was 50 years (interquartile range IQR: 45–58), 75% were men and the median time since HIV diagnosis was 14.5 years (IQR: 6.8–21.2). Overall, 91% of patients had received cART, 70% had less than 500 copies/mL at the latest plasma HIV RNA measurement, and the median CD4+ lymphocytes count within 6 months before death was 243 cells/mm^3^ (IQR: 91–451). HCV antibodies and hepatitis B surface antigen were detected in respectively 30% and 13% of the patients. Smoking and excessive alcohol consumption (more than 30 grams/day) were noticed in 71% and 25% of the cases, respectively.

The main underlying causes of death were AIDS (25% in 2010 versus 36% in 2005 and 47% in 2000), NANH-related cancers (22% versus 17% in 2005 and 11% in 2000), hepatic diseases (11% versus 15% in 2005 and 13% in 2000), cardiovascular diseases (10% versus 8% in 2005 and 7% in 2000), non-AIDS defining infections (9% versus 4% in 2005 and 7% in 2000) and suicide (5% versus 5% in 2005 and 4% in 2000) [12].

**Fig 1:**
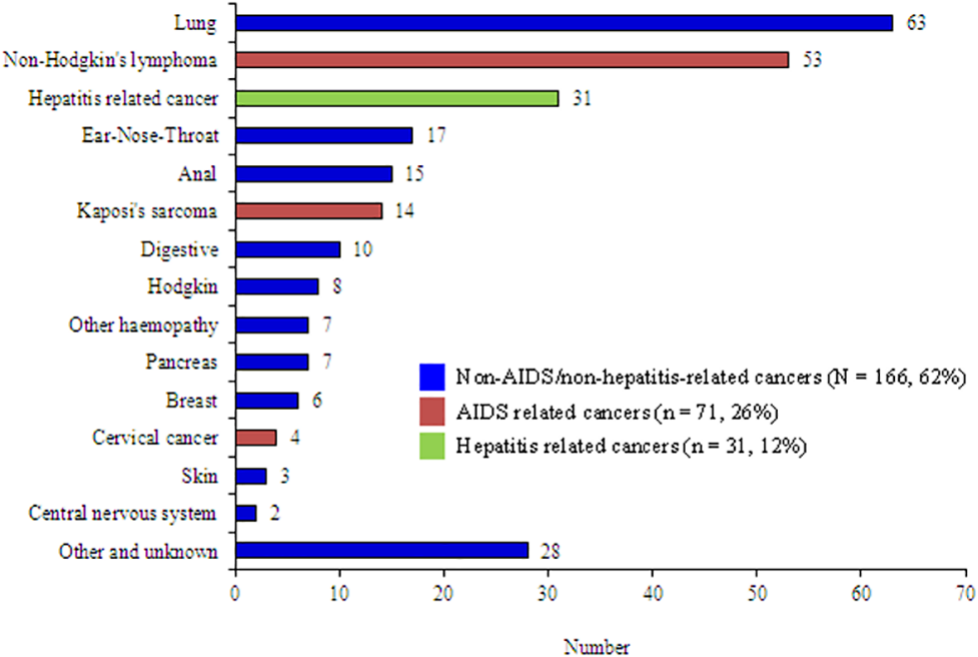
Location of cancers (N = 268) among HIV-infected adults with underlying cause of death being cancer (N = 262), Mortalité 2010 survey, France.

Among the 728 cases with information provided in 2010, 268 malignancies were deemed responsible for the death of 262 patients, representing 36% of all deaths (Fig 1). Cancers-related causes of deaths were distributed as follow (Table 1): AIDS-related cancers (N = 71 in 68 patients, 9% of the causes of death), hepatitis-related cancer (N = 31, 4%) and NANH-related cancers (N = 166 in 163 patients, 22%). The main patients' characteristics according to the underlying cause of death are reported in Table 2. The three major lethal morbidities were lung cancer (n = 61), NHL (n = 53) and hepatocarcinoma (n = 31).

**Table 1:** Cancer-related causes of death.

	Mortalité 2000	Mortalité 2005	Mortalité 2010	p-value^a^
**Reported deaths**	**964**	**1042**	**728**	
**Cancer-related causes of death, n (%)**	**269 (27.9%)**	**344 (33.0%)**	**262 (36.0%)**	**0.003**
**AIDS-related, n (%)**	**149 (15.5%)**	**134 (12.9%)**	**68 (9.3%)**	**0.024**
Non Hodgkin lymphoma	105 (10.9%)	103 (9.9%)	53 (7.3%) ^b^	0.122
Kaposi sarcoma	40 (4.1%)	25 (2.4%)	11 (1.5%)	0.084
Cervical cancer	4 (0.4%)	6 (0.6%)	4 (0.5%)	0.848
**Hepatitis-related, n (%)**	**17 (1.8%)**	**37 (3.6%)**	**31 (4.3%)**	**0.028**
Hepatitis C	8 (0.8%)	27 (2.6%)	19 (2.6%)	0.021
Hepatitis B	7 (0.7%)	6 (0.6%)	10 (1.4%)	0.279
Hepatitis B and C	2 (0.2%)	4 (0.4%)	2 (0.3%)	0.732
**Non AIDS/non hepatitis related, n (%)**	**103 (10.7%)**	**173 (16.6%)**	**163 (22.4%)**	**<0.001**
Respiratory	50 (5.2%)	65 (6.2%)	78 (10.7%)	0.004
Lung	44 (4.6%)	53 (5.1%)	61 (8.4%)	0.040
Ear, nose and throat	6 (0.6%)	12 (1.2%)	17 (2.3%)	0.056
Digestive	6 (0.6%)	13 (1.2%)	10 (1.4%)	0.342
Pancreas	3 (0.3%)	11 (1.1%)	7 (1.0%)	0.282
Anal	6 (0.6%)	11 (1.1%)	13 (1.8%)	0.073
Skin	2 (0.2%)	10 (1.0%)	3 (0.4%)	0.065
Hodgkin’s lymphoma	12 (1.2%)	9 (0.9%)	8 (1.1%)	0.473
Other hemopathies	5 (0.5%)	8 (0.8%)	7 (1.0%)	0.602
Breast	3 (0.3%)	7 (0.7%)	5 (0.7%)	0.647
Central nervous system	4 (0.4%)	6 (0.6%)	2 (0.3%)	0.530
Other and unknown^c^	12 (1.2%)	33 (3.2%)	27(3.7%)	0.029
Multiple^d^	-	-	3 (0.4%)	-

The Mortalité 2000, Mortalité 2005 and Mortalité 2010 surveys, France.

^a^Comparisons between 2000, 2005 and 2010 adjusted on age and gender

^b^including 3 patients with both non Hodgkin lymphoma and Kaposi sarcoma

^c^See Appendix for details

^d^Multiple: anus+prostate, anus+lung, lung+breast

**Table 2:** Patients' characteristics, by underlying cause of death–Mortalité 2010 survey, France.

	Non-Hodgkin Lymphoma^a^	Hepatitis-related cancers	Non-AIDS/non-hepatitis cancers	Non-cancer deaths
	(N = 50)	(N = 31)	(N = 163)	(N = 466)
Gender male (%)	78	87	79	77
Age (years, median [IQR])	48 [40-55]	51[46-55]	52 [47-60]	50 [44-58]
Time since HIV diagnosis (years, median [IQR])	4 [1-15]	19 [13-22]	16 [10-22]	15 [7-21]
CDC stage C (%)	100	32	48	61
CD4 count^b^ (cells/mm^3^, median, IQR)	75 [22-218]	269 [187-550]	287 [175-450]	244 [94–452]
CD4 ≥ 500 cells/mm^3^ (%)	0	26	17	19
Previous antiretroviral treatment (%)	94	97	96	92
ARV treatment duration (years, median [IQR])	2 [1–10]	13 [9-15]	12 [8-15]	12 [6-15]
HIV-RNA^b^ < 500 copies/mL (%)	58	90	89	70
Intravenous drug user (%)	9	61	30	34
Hepatitis C (%)	8	68	28	31
Hepatitis B (%)	4	39	14	15
Excessive alcohol consumption^c^ (%)	12	26	19	27
Smoking^d^ (%)	60	83	80	73

^a^excluding primary brain lymphoma

^b^last measure of CD4 or HIV-RNA before death

^c^ more than 30 grams/day

^d^current or past consumption

IQR: interquartile range; HIV: human immunodeficiency virus; AIDS: acquired immunodeficiency syndrome; ARV: antiretroviral

The proportion of deaths attributable to malignancies increase over time from 28% in 2000 to 33% in 2005 and 36% in 2010 (p = 0.003) (Table 1). The proportion of deaths attributed to AIDS-defining cancers decreased significantly from 2000 to 2005 and 2010 (16% to 13% to 9% respectively, p = 0.024), while the proportion of deaths attributed to hepatitis-related cancers slightly increased (from 2 in 2000 to 4% in 2010, p = 0.028). Those attributed to NANH-related cancers significantly increased during the same period (11% in 2000 to 17% in 2005 and 22% in 2010, respectively, p<0,001).

Patients who died from NANH-related cancers were 52 years old in median, (*versus* 49 years in 2005 and 46 years in 2000), 80% of them were smokers and 19% had excessive alcohol consumption (Table 2). The prevalence of smoking was significantly higher in patients who died of lung cancer compared to patients who died of other causes of cancer-related deaths, as 98% of them were smokers (p<0.001). They were moderately immunosuppressed (last median count within 6 months before death: 287 CD4/mm^3^ in 2010 versus 205 CD4/mm^3^ in 2005 and 218 CD4/mm^3^ in 2000), and 89% had less than 500 copies/mL of plasma HIV-RNA at the latest plasma HIV RNA measurement before death (61% in 2005). Ninety-six percent of them had been previously treated with antiretroviral drugs (90% in 2005 and 84% in 2000).

Lung cancers were the most frequent among lethal NANH-related cancers in 2010 and the most frequent among all lethal cancers instead of NHL so far (Fig 1), with a substantial increase in their proportion among all causes of deaths from 2000 to 2010 (5% in 2000 to 8% in 2010, p = 0.040). The proportion of lethal digestive and pancreatic cancers remained stable from 2000 to 2010. The proportion of lethal non-AIDS hemopathies, including Hodgkin’s lymphoma, remained also similar between 2000 and 2010.

Regarding AIDS-related malignancies, the proportion of NHL-related deaths trended to decrease between 2000 and 2010 (11% in 2000, 10% in 2005 and 7%, in 2010, p = 0.122). Patients with lethal NHL had a recent diagnosis of HIV infection (median duration of HIV infection: 4 years) and were highly immunosuppressed with CD4 cell count in the same magnitude than in the previous national surveys (median: 75 CD4/mm^3^ in 2010 *vs* 76 CD4/mm^3^ in 2005 and 86 CD4/mm^3^ in 2000), with a higher proportion of patients with HIV RNA <500 copies/ml (58% in 2010 vs 48% in 2005). The proportion of lethal cervical cancer and Kaposi sarcoma remained stable between 2000 and 2010.

At last, patients who died from hepatitis-related cancer were 51 years old in median. They were moderately immunosuppressed (median of 269 CD4 /mm^3^ in 2010 vs 231 CD4/mm^3^ in 2005 and 157 CD4/mm^3^ in 2000), and 90% had less than 500 copies/mL of HIV-RNA at time of death (69% in 2005). Patients who died from hepatocarcinoma were more often co-infected with hepatitis C virus than with hepatitis B virus (68% and 39% respectively, including 7% of patients co-infected with hepatitis B and C). Patients with hepatitis-related cancers were often exposed to excessive alcohol consumption (26%) and had history of intravenous drug use (61%).

## Discussion

In this large French prospective survey specifically designed to assess the distribution of the underlying cause of death in HIV-infected patients in 2010, we showed that cancers represented the leading cause of mortality in patients with HIV, accounting for more than one-third of the causes of death. Although a decrease of the proportion of deaths attributed to AIDS-related cancers, the proportion of death attributed to malignancies increased significantly since 2000, due to the slight increase of hepatitis-related cancers and mainly the dramatic increase of the proportion of deaths attributed to NANH-related cancers.

NANH-related cancers were the main part of lethal malignancies (62%), and represented a substantial increasing cause of death (from 11% in 2000 to 22% in 2010), and lung cancer was the most common cancer-related cause of death in 2010 instead of NHL so far. Other recent papers report an increase in the proportion of deaths due to NANH-related cancers and a progressive decrease in the proportion of deaths due to AIDS-defining cancers, including NHL [3,15–18]. While overall death rate trends to decrease since 2000, the rate of NANH cancer-related death remained constant over time and is now the most common non-AIDS cause of death in high-income countries [19].

The NANH-related cancers appeared to have a poor prognosis with a high prevalence of HIV patients with an advanced stage cancer at the diagnosis [20,21]. Especially, lung cancer seemed to have an earlier onset, more advanced stage and worse prognosis in HIV-infected patients than in the general cancer population [6–8]. This young age of onset and advanced clinical stage might reflect more aggressive cancers in HIV-infected patients although the higher prevalence of traditional risk factor such as smoking in this population has an important impact. While HIV infected patient with Hodgkin lymphoma had more extensive disease, the prognosis seems to be now the same than the general population [22,23].

Early detection of lung cancer is the predominant driver of survival. There are currently not enough data to recommend for or against wide-spread screening for lung cancer with low-dose computed tomography scan (LDCT) in asymptomatic HIV patients. In the general population, American College of Chest Physicians (ACCP) suggested recently than annual screening with LDCT should be offered for smokers aged 55 to 74 who have smoked at least for 30 pack-years [24]. Screening programs for lung cancer with LDCT might be warranted for HIV-infected smokers, as most of these cancers are discovered at a late stage, when curative treatment is no longer available. It should be also discussed to screen HIV-infected smokers earlier than smokers of the general population due to the younger age at the diagnosis of cancer in this population. Given the frequency of pulmonary diseases in HIV-infected patients, the false-positive rate of LDCT screening might however be higher than in the general population (and so associated risks and costs due to follow-up CT scans and potentially more invasive procedures). The interest and cost-effectiveness of lung cancer screening with LDCT are presently being evaluated in a French study (ANRS EP48 HIV CHEST, http://www.clinicaltrials.gov NCT01207986).

It must be emphasized that the most important contributor to reducing lung cancer-related mortality is to quit smoking. Lung cancer incidence and mortality appears to be higher in HIV infected patients than the general population, independently of smoking [25,26]. Nevertheless, smoking represents the main independent risk factor in the development of lung cancer in HIV infected patients [9]. Higher smoking rates are reported in this population than in the general population [25] with a prevalence around 50% in European HIV cohorts [27,28] compared to 27% in the general population of comparable age and gender [29]. In our study, 98% of the patients who died of lung cancer were smokers. Smoking cessation could reduce the risk of lung cancer mortality by more than 50% [30]. Patients who are smokers must be counseled aggressively for smoking cessation and treated for tobacco addiction, and smoking cessations programs in HIV population must be a priority.

If the proportion of NHL-related death tended to decrease slightly (from 11% in 2000 to 7% in 2010), this remains a significant proportion in a high-income country with high access to health care and cART. Patients with NHL as underlying cause of death had often a recent diagnosis of HIV infection and were highly immunosuppressed. Improving screening for HIV infection in order to diagnose infected individuals at an earlier stage and starting cART in all HIV-infected patients should lead to improve immune system and may then allow to further decrease deaths due to NHL-related cancers [17,31]. Patients highly immunosuppressed remain to be closely screened for AIDS-defining cancers.

After an increase in deaths related to hepatitis-relative cancer between 2000 and 2005, stabilization was observed between 2005 and 2010, and mortality due to hepatitis-related cancer should decline in the near future with improvement of hepatitis C treatment. Early detection of hepatocarcinoma and avoiding excessive alcohol consumption remain a crucial point in the management of patients co-infected with viral hepatitis.

The increasing proportion of lethal non-AIDS-related cancers may be related first to the improved life expectancy and so the aging of this subpopulation [32], the reduction of competing causes of death, but also still a higher prevalence than the general population of traditional major risk factors of cancers (such as tobacco and alcohol use). In our study, patients with lethal non-AIDS-related cancers were moderately immunosuppressed (median CD4 cell count at 287 cells/mm^3^, despite an increasing proportion of patients with a HIV RNA <500 copies/ml (from 61% in 2005 to 89% in 2010). A meta-analysis comparing the incidence of cancers in HIV-infected persons and immunosuppressed transplant recipients has strongly suggested a link between immunosuppression and cancer [33]. Several studies have reported a strong association of immunosuppression even moderate (CD4 cell count <350 cells/mm^3^ [34] and even <500 cells/mm^3^) [9,35] with higher risk of non-AIDS-related cancers [31,36], supporting arguments for earlier initiation of cART in order to reach CD4 lymphocytes levels as high as possible to reduce the risk of cancer-related death.

These findings highlight first of all the importance of preventing risk factors (such as smoking and excessive alcohol consumption cessation, prevention and treatment of hepatitis B and C viral infections). Preventing immunosuppression by starting cART as soon as possible may also reduce the risk of cancer-related death. These results also underline the importance of promoting more efficient screening strategies to allow earlier diagnosis of cancer in HIV patients. Besides, they justify a close collaboration between oncologists and HIV specialists in order to optimize the management and the outcome of these patients, taking into account immune status, prophylactic measures and potential interactions and/or cumulative toxicities between chemotherapy and antiretroviral treatment [37]. Optimal cancer management, similar to that of the general population, should be offered to HIV-infected patients. Moreover, the high incidence of cancer in HIV infected people justifies that systemic HIV testing should be performed as routine care in all cancer patients [38,39].

This study is a unique nation-wide survey which described the underlying cancer-related causes of death among patients living with HIV in France in 2010 and their evolution over a 10-year period, in a country where all HIV-infected patients have free access to care and all treatments are provided free of charge, including for migrants. Although the 2010 survey was conducted only in the centres reporting at least five deaths during one of the previous surveys, patients in care in these centres represented almost 75% of the entire HIV infected population estimated to be in care in France in 2010 and 55% of all HIV infected patients living in France, including those who ignore their diagnosis [12]. Thus, we believe that the Mortalité 2010 study provides an accurate estimation of the causes of death including cancers in high income countries given the process of documentation and classification performed (expertise from two previous similar studies, detailed standardized questionnaire, coherence of data validated by two experienced physicians and role of the national reference Centre on Medical Causes of deaths).

Besides, we acknowledge some limitations of our study. This study was designed to assess the distribution of the underlying cause of death in HIV-infected patients, and then did not allow us to provide data upon the mortality rate of cancer-related death. Moreover, this study was a descriptive transversal study that did not allow us to determine prognostic factors for cancer-related causes of death.

In conclusion, an increasing proportion of deaths attributable to non-AIDS-related cancers was observed among HIV-infected patients, and lung cancer was the leading cancer-related cause of death in 2010 instead of NHL so far. Cancer prevention especially smoking cessation programs, screening strategies for early detection of cancer, and therapeutic management need to be optimized in HIV-infected patients in order to reduce mortality. HIV testing should be routinely offered to all cancer patients.
